# Improving GNSS PPP Convergence: The Case of Atmospheric-Constrained, Multi-GNSS PPP-AR

**DOI:** 10.3390/s19030587

**Published:** 2019-01-30

**Authors:** John Aggrey, Sunil Bisnath

**Affiliations:** Department of Earth and Space Science and Engineering, York University, Toronto, ON M3J 1P3, Canada; sbisnath@yorku.ca

**Keywords:** GNSS, Precise Point Positioning, Global Ionospheric Maps, ionosphere, troposphere, convergence

## Abstract

GNSS positioning performance has been shown to improve with the ingestion of data from Global Ionospheric Maps (GIMs) and tropospheric zenith path delays, which are produced by, e.g., the International GNSS Service (IGS). For both dual- and triple-frequency Precise Point Positioning (PPP) processing, the significance of GIM and tropospheric products in processing is not obvious in the quality of the solution after a few hours. However, constraining the atmosphere improves PPP initialization and solution convergence in the first few minutes of processing. The general research question to be answered is whether there is any significant benefit in constraining the atmosphere in multi-frequency PPP? A key related question is: regarding time and position accuracy, how close are we to RTK performance in the age of multi-GNSS PPP-AR? To address these questions, this paper provides insight into the conceptual analyses of atmospheric GNSS PPP constraints. Dual- and triple-frequency scenarios were investigated. Over 60% improvement in convergence time was observed when atmospheric constraints are applied to a dual-frequency multi-GNSS PPP-AR solution. Future work would involve employing the constraints to improve low-cost PPP solutions.

## 1. Introduction

Traditionally, the effects of ionosphere and troposphere refraction are mitigated in the Global Navigation Satellite System (GNSS) Precise Point Positioning (PPP) measurement processing technique using dual- or triple-frequency linear combinations and systematic modelling, respectively [1,2,3,4,5]. The purpose of such mitigation hinges on improving PPP convergence and initialization which has been a challenging but promising area of GNSS research. Recent developments and contributions highlight the changing definition of conventional PPP from the use of dual- to triple-frequency measurements. Additional frequencies coupled with expanding satellite constellations have improved reliability and integrity of multi-GNSS PPP solutions [6,7,8,9]. Thus, the progression towards the improvement of multi-GNSS PPP solution quality and initial convergence is only natural given that measurement strength and satellite geometry are continually being enhanced.

It is well known that by accessing the slant ionospheric information, PPP convergence and initialization can be significantly improved [10,11,12,13]. The usage of raw observations in an uncombined PPP processing is gradually becoming the standard approach as an alternative to ionosphere-free PPP solutions. One key advantage involves the flexibility in processing the observations from all existing GNSS constellations while avoiding noise amplification due to linear combinations [14,15,16]. The consequential benefit is the ability to extract the slant ionospheric delays which can be used to quickly re-initialize the solution. In the context of this paper, the raw observations from available GNSSs were processed without employing linear combinations to eliminate the first order ionospheric delay, but rather estimate it. Having access to the slant ionospheric terms enable various analyses to be made in comparison to the a priori ionospheric delays obtained through atmospheric products. For more and detailed information on how to decompose the linear ionospheric combination to its uncombined format in order to estimate the slant ionospheric term, the reader is referred to these research contributions that discusses it at length: [17,18,19,20].

Using a priori estimates from atmospheric products, either from regional or global networks of stations, it has been shown that it is possible to obtain cm-level accuracy within a few minutes, rather than the typical 20 to 30 min of PPP convergence time [17,21,22,23,24,25]. Single-frequency ambiguity resolution (AR) has been a prominent topic demanding innovative solutions due to its relatively long convergence time period [14,26,27,28]. Isolating and resolving ambiguities to integers can be hastened by using a priori atmospheric constraints, which consequentially leads to improved accuracy and stability in the position estimates [17,18,22].

The purpose of this study is to demonstrate how close multi-GNSS PPP performance is to RTK-like performance by focusing on the increasing improvements seen in the reduction of convergence time. As it is well-known, typical RTK performance is correlated and limited to baselines of less than approximately 20 km. Longer baseline lengths prevent measurement errors from being effectively accounted for. By mathematically differencing GNSS measurements from multiple receivers, the key objective of relative positioning is to reduce or eliminate error sources. One key benefit of PPP is that it removes the need for a local GNSS network and allows for centimetre- to millimetre-level positioning accuracy with a single GNSS receiver. The disadvantage of conventional PPP is the limitation of needing tens of minutes to obtain centimetre-level positioning, as defined in terms of convergence period. Similarly, when considering typical RTK positioning performance, the potential delocalization of the ionosphere and troposphere introduces systematic errors that prevent baselines from being extended beyond 20 km if reliable user solutions are to be obtained [29]. As PPP accuracy is independent of baseline length, improving its convergence and performance is necessary if it is to be considered as a good alternative to RTK technique. The scope of this paper therefore addresses atmospheric constraining in PPP with the aim of improving the solution accuracy and quality.

There is a level of progression which can be noticed from the age of dual-frequency multi-GNSS processing through the usage of triple-frequency measurements to the application of atmospheric constraints. Specifically, the following key questions are to be answered: (1) What is the magnitude of improvements observed from the usage of traditional dual-frequency measurements to triple-frequency PPP processing with atmospheric constraints? (2) What are the inherent challenges when constraining PPP solutions with atmospheric corrections either functionally or stochastically? (3) What is the significance of PPP-AR in multi-GNSS PPP atmospheric constrained solution? (4) Finally, what are the key challenges left in obtaining near-instantaneous PPP convergence akin to RTK data processing?

## 2. Datasets Used and Data Processing Details

**Figure 1:**
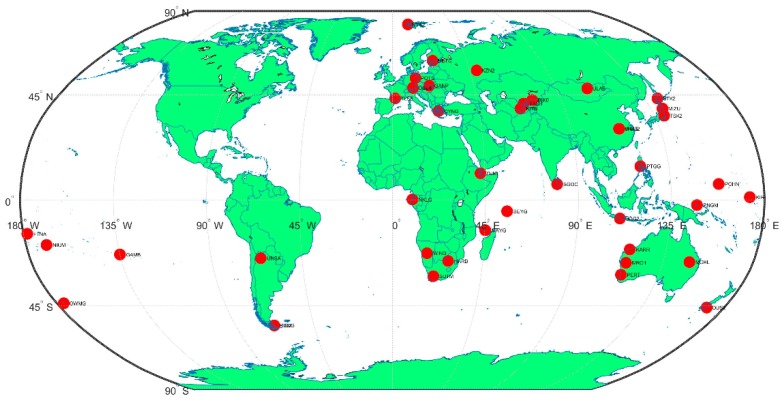
Map of global distribution of selected multi-GNSS stations.

To investigate the impact of atmospheric parameter constraints in both dual- and triple-frequency multi-GNSS PPP, 40 globally distributed MGEX GNSS receiver stations were arbitrarily selected for PPP processing. Figure 1 shows the global distribution of stations.

Observations from these stations were processed from DOY 253 to 259 in 2018. The stations were randomly selected based on homogeneity of receiver and antenna types to limit the effects of equipment hardware delays impacting the results. Javad (San Jose, CA, USA) and Trimble (Sunnyvale, CA, USA) receiver-antenna combinations were considered.

Shown in Table 1 are the processing parameters and settings used in the generation of results discussed in subsequent sections. The YorkU GNSS PPP engine, developed by the GNSS research team in York University (Toronto, Canada), was employed in processing the observations [30,31]. GBM precise orbits and clocks from GeoForschungsZentrum (GFZ, Potsdam, Germany) were used because of the availability of orbits and clocks for all available GNSS constellations.

## 3. Concepts of Functional and Stochastic Atmospheric Constraints

Atmospheric products such as Global Ionospheric Maps (GIM) and zenith path delays might play an important role with regards to significant improvement in multi-GNSS PPP solution quality and accuracy. Using the a priori vertical total electron content (VTEC) and accuracies provided in GIM products, PPP convergence can be significantly improved. However, the positional accuracy can be affected given different approaches used in generating the GIM models, as well as the density of stations used.

### 3.1. Existing Atmospheric Products

#### 3.1.1. Existing GIM Products

Dual-frequency GNSS observations from ground networks are used to generate ionospheric delay models which are useful for both precise GNSS positioning and ionosphere study. Utilizing global or regional network of stations, ionosphere delay models generated are dependent and correlates with the scope of coverage of the reference networks. GIM is a typical example and is in the form of spherical harmonic functions [32]. The assumption made is that the electron density of the atmosphere is concentrated on an atmospheric layer at a fixed height, usually 350 to 450 km, in the global model recovery. Regarding this assumption, the slant ionospheric delays are expressed by a combination of the VTEC and a mapping function. The VTEC is represented by the estimations of the coefficients of the spherical harmonic function. The VTEC is then mapped to obtain the slant ionospheric delay through a mapping function after the ionosphere pierce point (IPP).

Over the past two decades, VTEC estimates and their associated rms estimates, have been provided, originally for meteorological purposes, to the GNSS community through GIMs. As part of the estimation process, satellite and receiver differential code biases (DCBs) with their rms values, are provided as a by-product. An average of 21 GIM products currently exist. These products are either post-processed, predicted or combined from other analysis centres (ACs) and generated from dual-frequency GNSS measurements. Shown in Table 2 are the unique current products available to GNSS users. Of interest is the varying numbers of receiver stations and GNSSs used in the generation of the products. 

**Figure 2:**
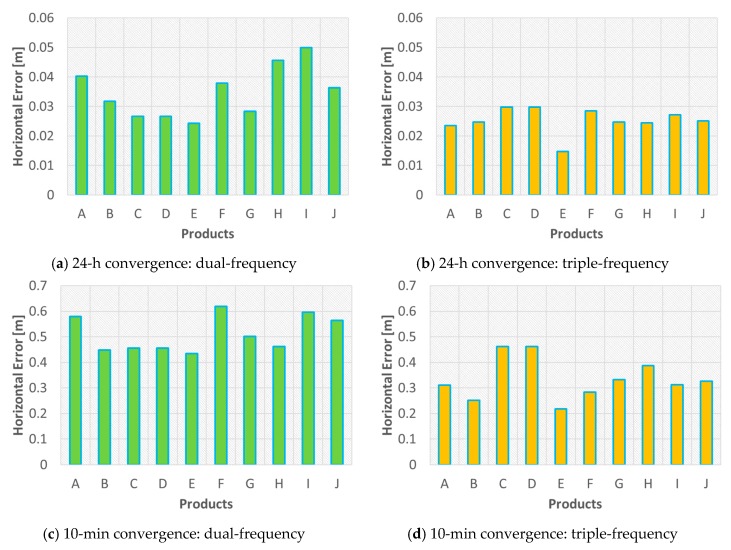
Analyses for 24-h and 10-min convergence for different existing GIM products using 3 days of 24-h observations from MGEX multi-GNSS stations.

Figure 2 shows the results of how different current GIM products affect the performance of ionospheric constraining in multi-GNSS PPP solutions. Using 3 days of 24-h observations from MGEX multi-GNSS stations mapped out in Figure 1, solutions were obtained for both dual- and triple-frequency PPP processing modes. It must be pointed out that only post-processed products were used for the sake of uniformity – the rationale being to demonstration whether after 24 h of processing, PPP convergence is affected by the kind of product used, as outlined in Table 2. A secondary objective, where ionospheric constraining plays a major role, PPP convergence in relation to the horizontal error was investigated for the first 10 min using the different GIM products. As observed in Figure 2a,b, over 24 h, there are millimetre-level variations between the products in the dual- and triple-frequency scenarios, respectively.

A similar pattern was observed in the first 10 min of PPP convergence, as depicted in Figure 2c,d. However, in the 10-min scenario, centimetre-level differences could be seen, though some of the products still had millimetre range differences. These differences were observed in both the dual- and triple-frequency PPP results. It must be clarified that the purpose of these analyses was not to pitch or promote one product against another but rather highlight the influence that the choice of product could have on PPP initial solution convergence. It is important to point out that the GIM product for a particular day represents a snapshot of the ionospheric activity occurring within that period of time. Hence, the results shown are not conclusive for all other days, as each day can potentially show varying results from those presented for DOY 253 to 259 in 2018. Only 10 post-processed GIM products were investigated given the fact that it was difficult to obtain all the existing products from archiving sources. However, the key intended point is to show that depending on the product used to constrain PPP solutions, especially in the first few minutes, there is the possibility of centimetre- to millimetre-level of differences that could be observed.

#### 3.1.2. Zenith Path Delay Products

Using ground-based GNSS information, tropospheric products are generated from IGS ACs. Horizontal gradient components and 5-min interval estimates of zenith path delay (ZPD) are included in the products. Using over 350 IGS GNSS stations, ZPD estimates are generated and made available daily per site. Precipitated water vapour extracted from estimated ZPDs are included in the products due to surface pressure and temperature measurements made at GNSS site locations. Given that the tropospheric products are generated using IGS final satellite orbits and Earth Orientation Parameters (EOP) products, they are therefore available approximately three weeks after the day of observation. Different methodologies are employed in the generation of the IGS combined tropospheric solution which potentially impacts the accuracy of the products. The production and dissemination of these products since inception has been the focus of three ACs, namely the Jet Propulsion Laboratory (JPL, Pasadena, CA, USA), German Research Centre for Geosciences (GFZ) and United States Naval Observatory (USNO, Washington, DC, USA) [34,35,36,37,38,39].

### 3.2. Methods of Constraint Application

Though not a novel concept, it is well known that constraining an estimable parameter to a known value in a least squares or Kalman filter might aid in the optimization of the estimated solution. Applying atmospheric constraints functionally implies fixing the atmospheric parameters in the functional pseudorange and carrier-phase models. Equation (1) illustrates the well-known models with the parameters to be constrained:

(1) $$\begin{array}{l} P_{r}^{s}=\rho_{r}^{s}+c(dt_{r}-dt^{s})+T_{r,constrained}^{s}+I_{r,constrained}^{s}+\varepsilon_{P}\\ \phi_{r}^{s}=\rho_{r}^{s}+c(dt_{r}-dt^{s})+T_{r,constrained}^{s}-I_{r,constrained}^{s}-\lambda N_{r}^{s}+\varepsilon_{\phi } \end{array}$$

where $P_{r}^{s}$, $\phi_{r}^{s}$ denote the pseudorange and carrier-phase observations from satellite (*s*) to receiver (*r*), respectively; $\rho_{r}^{s}$ is the geometric range between the satellite and receiver antennas; *dt_r_* and *dt^s^* represent the receiver and satellite clock errors, respectively; $T_{r,constrained}^{s}$ refers to the constrained tropospheric delay; $I_{r,constrained}^{s}$ is the constrained ionospheric delay on the GNSS signal propagated; *λ* is the wavelength; $N_{r}^{s}$ is the carrier-phase ambiguity including satellite and receiver phase instrumental delays and initial fractional phase bias; *ε_P_* and *ε_φ_* refer to a combination of observation noise, satellite and receiver instrumental delay s due to the transmitting and receiving hardware, multipath and unmodelled effects, respectively. By constraining atmospheric parameters in the functional models, the partial derivatives of these parameters, represented by the design matrix, must be deleted. Though this action eliminates the need to estimate the constrained parameters by using atmospheric products, there is the potential of residual bias in the solution due to time correlated errors. 

An simpler option presents itself through stochastic constraints. Using the ionospheric delay and tropospheric estimates, as well as their uncertainties provided in the atmospheric products, the covariance matrix can be adjusted to constrain the atmospheric parameters. It is key to note that there is no need to constrain every epoch given the potential of propagating time correlated errors. By constraining only the first epoch and “freeing” up the process noise to allow the filter to continue estimating the atmospheric parameters, the constraint initially applied should have a beneficial effect in the first few epochs, improving the solution accuracy in PPP processing. In a sequential least-squares implementation, updating and propagating the covariance information, $C\hat{x}$, the first epoch requires the inclusion of appropriate process noise, $C\varepsilon_{\Delta t}$, assuming a time interval of Δ*t*. This process can be represented by:

(2) $$Px^{0}={\left[C\hat{x}+C\varepsilon_{\Delta t}\right]}^{-1}$$

$C\hat{x}$, representing a priori variance-covariance matrix, is given by:

$$C\hat{x}=\left[\begin{array}{cccc} \sigma_{x}^{2} & & & \\ & \sigma_{y}^{2} & & \\ & & \sigma_{z}^{2} & \\ & & & \begin{array}{cccc} \begin{array}{c} \ddots \end{array} & \begin{array}{c} \begin{array}{c} \sigma_{ZPD,constrained}^{2} \end{array} \end{array} & \begin{array}{c} \begin{array}{c} \begin{array}{c} \ddots \end{array} \end{array} \end{array} & \begin{array}{c} \begin{array}{c} \begin{array}{c} \sigma_{iono,constrained}^{2} \end{array} \end{array} \end{array} \end{array} \end{array}\right]$$

$C\varepsilon_{\Delta t}$, defined as the process noise covariance matrix, is also given by:

$$C\varepsilon_{\Delta t}=\left[\begin{array}{cccc} C\varepsilon {\left(x\right)}_{\Delta t} & & & \\ & C\varepsilon {\left(y\right)}_{\Delta t} & & \\ & & C\varepsilon {\left(z\right)}_{\Delta t} & \\ & & & \begin{array}{cccc} \begin{array}{c} \ddots \end{array} & \begin{array}{c} \begin{array}{c} C\varepsilon {\left(ZPD\right)}_{\Delta t,constrained} \end{array} \end{array} & \begin{array}{c} \begin{array}{c} \begin{array}{c} \ddots \end{array} \end{array} \end{array} & \begin{array}{c} \begin{array}{c} \begin{array}{c} C\varepsilon {\left(iono\right)}_{\Delta t,constrained} \end{array} \end{array} \end{array} \end{array} \end{array}\right]$$

It is imperative to note that the choice of process noise value, as well as atmospheric constraint uncertainty, affects the effectiveness of the filtered solution in the first epochs of processing. To avoid the case of over-constraining, the uncertainties of the GIM delays should be inflated to accommodate any inherent errors in the generation of these ionospheric delays. Concurrently, and in the same vein, it is necessary to choose an appropriate process noise which does not dilute the impact of the uncertainties used as constraints. In other words, a large process noise could lead to under-constraining, while a small process noise value potentially over-constrains depending on the GIM delay uncertainty.

Similarly, the ZPDs with standard deviations provided in tropospheric products, can be used in constraining the atmosphere in multi-GNSS PPP solutions. The reason for the addition of a ZPD constraint, considering a sequential least-squares adjustment, is to either improve PPP convergence in the first few minutes or to avoid matrix inversion singularities, given poor satellite geometry. A fixed ZPD constraint of 20 cm was used for analyses in the results presented for the multi-GNSS PPP processing in this paper. Given that the ZPD products are made available daily by GNSS site, the choice of a fixed constraint stems from the fact that products were not available for majority of the stations analyzed. Hence, the constraint was empirically chosen based on the consistency of the estimates obtained from the few products made available.

## 4. Variations between Estimated Slant and GIM Delays

**Figure 3:**
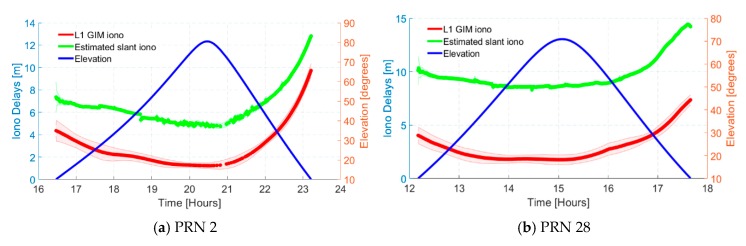

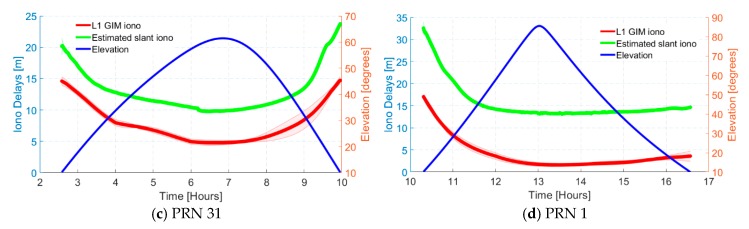
Magnitude of L1 GIM and estimated slant ionospheric delays for sample satellites with metre-level variations shown against satellite elevations. Delays are shown for DOY 253, 2018 for GMSD station, located in Japan. Bordered shaded colours represent the uncertainties.

A combination of the VTEC and a mapping function express the slant ionospheric delays generated in GIM. The differences between GIM and estimated slant ionospheric delays for particular satellites are depicted in Figure 3. As observed in Figure 3a–d, different satellites show metre-level variations between GIM and slant delays in PPP processing, especially within the first few minutes. These observed variations are not only due to different ionospheric estimates but there is the potential of unmodelled biases [40,41]. These differences were observed to be consistent for all available GNSSs.

In comparing the GIM and estimated slant delays, a key question presents itself: considering PPP float and fixed solutions, how precise must the GIM be in order to notice significant improvement in atmospheric constrained solutions? Unfortunately, attempting an answer represents a meander rather than a straight trajectory. Aside from being multi-faceted, the answer is reliant on the method and models used in the GIM generation (product-wise), how the GIM is used in PPP processing (application-wise), and quality of other GNSS products and observations (quality check). However, there is a level of consistency in the general trend of convergence for both slant and GIM delays. The bordered colours represent the uncertainties of GIM and slant delays, depicting the accuracy of the delays. As expected, given the convergence of the uncertainties of GIM delays, PPP solutions tend to become more optimistic. In the first few minutes of typical processing, a GIM is beneficial in PPP ionospheric constraining while the noisy pseudorange measurements are the limiting factor in accurately estimating slant ionospheric delays.

**Figure 4:**
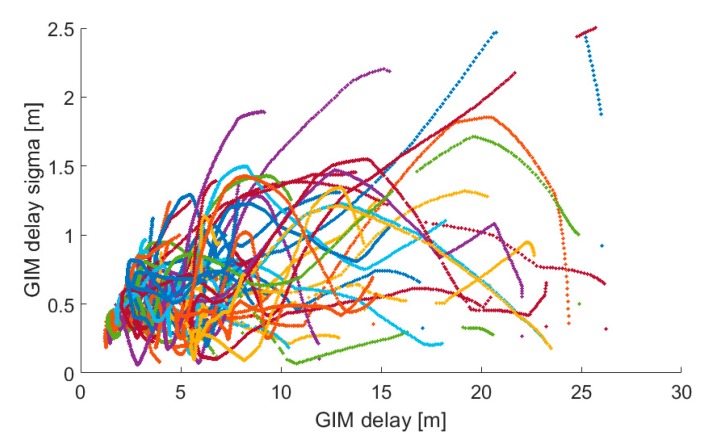
Correlation between GIM delays and their sigmas. Correlation coefficient equals 0.52. Each colour marker point represents a particular satellite. Delays are shown for DOY 253, 2018 for GMSD station, located in Japan.

**Figure 5:**
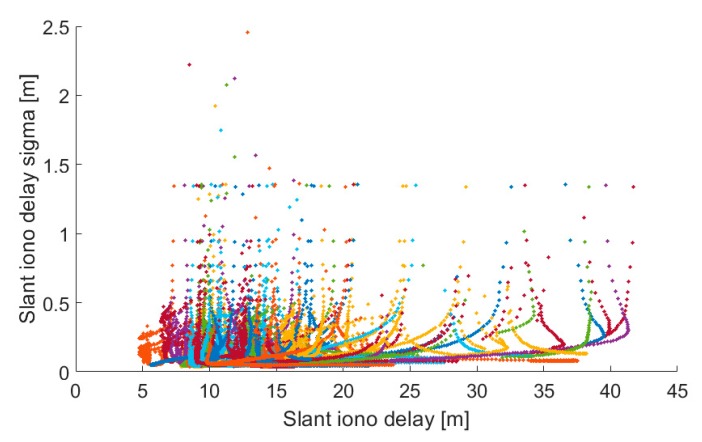
Correlation between estimated slant delays and their sigmas. Correlation coefficient equals 0.19. Each colour marker point represents a particular satellite. Delays are shown for DOY 253, 2018 for GMSD station, located in Japan.

Shown in Figure 4 and Figure 5 are correlations plots of GIM and estimated slant delays with their respective uncertainties. The correlation between the GIM delays and uncertainties range up to 8 m +/−1 m with a correlation coefficient of 0.52. This performance is in contrast to the estimated slant delays which ranged up to 10 m +/−0.3 m. while having a very weak correlation of 0.19.

The pivotal message from these analyses is that the critical minutes during PPP initialization show GIM with realistic ionospheric delays but decimetre-level uncertainties in contrast to the centimetre-level uncertainties for estimated slant delays. The typical slant ionospheric delay is more precise but less accurate, whereas GIM is more accurate but less precise in the first few minutes. This phenomenon elicits the need for an adaptive approach when using either GIM or ZPD product in constraining. The realism of the estimates and uncertainties is key in constraining. Unrealistic estimates and uncertainties could result in either over or under constraining. The observed product/measurement/processing sensitivity prompts urgency for further investigation to achieve an adaptive approach, which will be fully dependent on the GNSS measurements and parameters of the station, rather than just the atmospheric delays and uncertainties.

## 5. Dual- and Triple-Frequency Multi-GNSS PPP Atmospheric Constraining with AR

This section presents analyses on the impact of atmospheric constraints in multi-GNSS PPP processing. The purpose is to outline the progression of improvement in the usage of atmospheric constraints. While the aim is to improve the solution quality and accuracy, further insight is also provided on how different processing modes are impacted by atmospheric constraining. It must be noted that post-processed products are used for the analyses presented in this section. These post-processed products include the orbits and clocks as well as GIM. Real-time analyses were not considered in this paper given the unavailability of real-time GIM delays and ZPD estimates. 

### 5.1. Dual-Frequency Analysis

**Figure 6:**
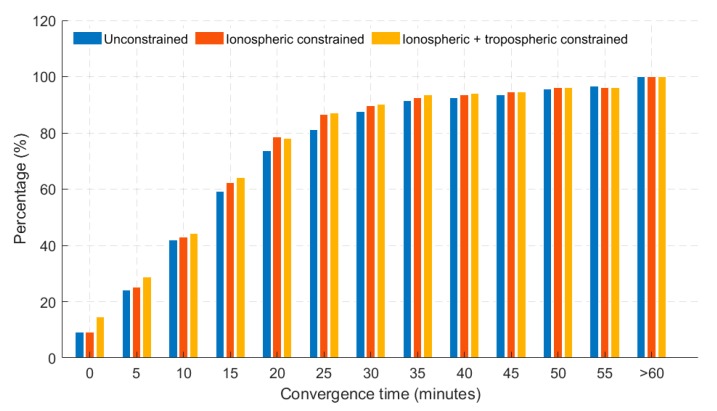
Histogram showing percentage of 40 stations converging based on 24 hourly solutions in dual-frequency GREC processing mode. Results shown have a 20 cm horizontal error threshold.

**Figure 7:**
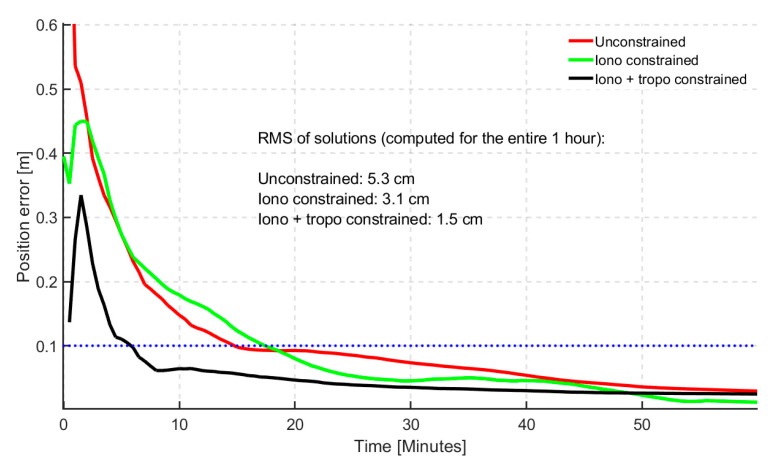
95th percentile time series showing horizontal positional error (hourly) based on 24 hourly solutions for 40 stations for dual-frequency GREC processing mode. Blue dotted line represents the 10 cm convergence threshold.

Shown in Figure 6 and Figure 7 are the histogram and time series of dual- frequency GPS(G) + GLONASS(R) + Galileo(E) + BeiDou(C) (GREC) PPP atmospheric-constrained, static horizontal solutions in the first hour of convergence, respectively. Defining convergence period as the time the solutions take to reach a horizontal error of 20 cm, the histogram shows a level of consistency benefiting PPP solutions when they are atmospherically constrained. Percentages of stations reaching the defined convergence threshold are shown. Three different scenarios were analyzed which included (1) unconstrained GREC PPP solutions, (2) Ionospheric (GIM) constrained and (3) GIM + tropospheric constrained GREC PPP solutions. It is interesting to observe that after 20 min, the differences between the solutions for GIM constrained and GIM + ZPD constrained solutions are minimal, indicating that optimal performance with GIM and ZPD constraints was obtained before the first 20 min. Constraining further after the 20 min-mark had very limited effect on improving positioning performance. This characteristic is understandable given that that atmospheric constraints are usually critical and effective only in the first few minutes of PPP convergence and initialization.

The prominent differences were observed within the first 15 min. The atmospheric constrained solutions saw an average of 6% improvement over the unconstrained, in terms of percentages of stations converging under 20 cm of horizontal error. However, the histograms do not tell the whole story. By observing the time series convergence period, the significance of the constrained solutions becomes even more obvious. By defining a stricter convergence of 10 min under a horizontal positional error of 10 cm, the 95th percentile of the atmospheric constrained solutions reached convergence in 6 min, as shown in Figure 7. The bump observed in the first few minutes could not be explained and will be investigated further. However, the effectiveness of applying atmospheric constraints is clearly seen in the first few epochs. The quickest initialization was observed for ionospheric and tropospheric constrained solution.

### 5.2. Triple-Frequency Analysis

**Figure 8:**
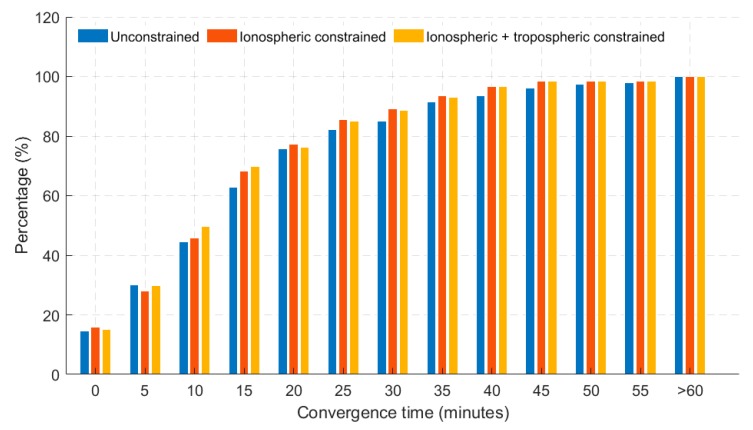
Histogram showing percentage of 40 stations converging based on 24 hourly solutions in triple-frequency GREC processing mode. Results shown have a 20 cm horizontal error threshold.

As shown in the dual-frequency scenario, similar characteristics were observed for the triple-frequency atmospheric constrained GREC PPP solutions. A look at the histogram, as shown in Figure 8, saw an average of 7% improvement over the unconstrained, in terms of percentages of stations converging under 20 cm of horizontal error. 

**Figure 9:**
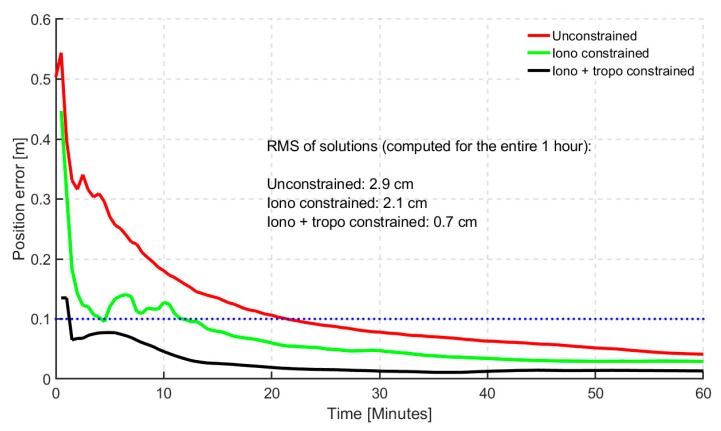
95th percentile time series showing horizontal positional error (hourly) based on 24 hourly solutions for 40 stations for triple-frequency GREC processing mode. Blue dotted line represents the 10 cm convergence threshold.

However, a significant level of improvement was observed by investigating the convergence of the time series of the atmospheric constrained solutions as depicted in Figure 9. Considering the 95th percentile and convergence defined as 10 min for solutions to fall under 10 cm of horizontal positional error, the atmospheric (iono + tropo) constrained solutions achieved convergence in 2 min. 

This level of improvement is significant and relevant as it reveals not only the benefits of having more frequencies, but how the coupling of these extra frequencies with atmospheric constraining can enhance the solution quality of multi-GNSS PPP solutions. In comparison to the dual-frequency constrained scenario, the triple-frequency constrained solutions had a 67% improvement in terms of solution convergence.

### 5.3. Dual-Frequency with GPS-AR Analysis

**Figure 10:**
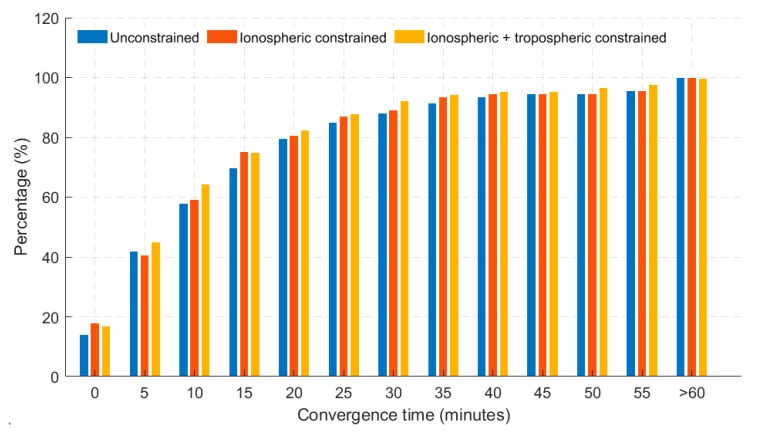
Histogram showing percentage of 40 stations converging based on 24 hourly solutions in dual-frequency GREC with GPS-AR processing mode. Results shown have a 20 cm horizontal error threshold.

**Figure 11:**
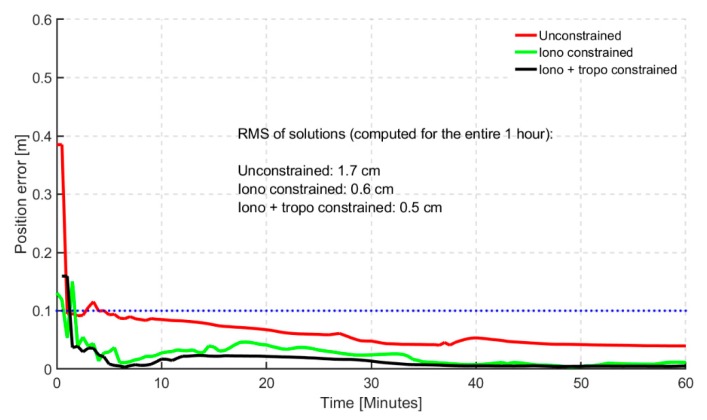
95th percentile time series showing horizontal positional error (hourly) based on 24 hourly solutions for 40 stations for dual-frequency GREC with GPS-AR processing mode. Blue dotted line represents the 10 cm convergence threshold.

The third scenario investigated involved resolving the ambiguities for GPS satellites while applying atmospheric constraints in a dual-frequency GREC PPP processing. Only GPS ambiguities were resolved for this analysis but future work would include GEC-AR. Similar to the other previous scenarios, the histogram, as shown in Figure 10, saw an average of 6% improvement over the unconstrained, in terms of percentages of stations converging under 20 cm of horizontal error. More insight was provided in the times series as shown in Figure 11.

With convergence defined as 10 min for solutions to fall under 10 cm of horizontal positional error, and considering the 95th percentile, the atmospheric constrained solutions with GPS-AR achieved convergence in less than 2 min. The steadiness of the converged solutions was consistent than triple-frequency atmospheric constrained solutions.

In summary, it is imperative to bring into context all the significant levels of improvement throughout all the various scenarios addressed in this paper. Shown in Table 3 are the significant improvements in convergence time observed with contributions from the research work in this paper. The typical convergence in minutes is compared to the research contributions through the results presented in this paper. With RTK-like performance being the target, it is expedient to address how close PPP is to RTK performance. Considering a typical RTK convergence of a few centimetres in a few minutes, the atmospheric constrained multi-GNSS PPP solutions presented is closely comparable to RTK performance, though we are not there just yet. The significance of this enhanced performance informs the possibility of using PPP in much more time-sensitive applications.

## 6. Conclusions and Future Work

Using GIM and tropospheric zenith delay corrections, a progression of improvements has been shown. Position accuracy and solution convergence were the key performance criteria assessed. By resolving ambiguities while constraining atmospheric parameters, it was observed that the multi-GNSS PPP solutions converged to a decimetre-level in less than 2 min for the horizontal components. Comparing the atmospheric constrained multi-GNSS PPP-AR to the unconstrained solution, a significant level of improvement was noticed which addressed the importance and efficacy of the constraints applied. The atmospheric constrained PPP solutions for triple-frequency PPP solutions showed more than 60% improvement in the position accuracy as compared to dual-frequency solutions. Using a strict convergence threshold of 10 min for the PPP solution to be steady under a horizontal error of 10 cm, the significance of atmospheric constraints in PPP-AR was shown. The realism of the GIM and estimated slant delays was also investigated which informs on the need to be cautious of either under or over constraining the PPP solutions. In summary, to address the original questions posed at the introduction of this contribution, here are the brief conclusions:

(1) What is the magnitude of improvements observed from the usage of traditional dual-frequency measurements to triple-frequency PPP processing with atmospheric constraints?
  Results presented show a significant level of improvement of more than 60% from atmospheric constrained dual- to triple-frequency multi-GNSS PPP in terms of the reduction in convergence time.
(2) What are the inherent challenges when constraining PPP solutions with atmospheric corrections either functionally or stochastically?
  It was shown that caution needs to be taken when considering using GIM estimates and their uncertainties to constrain. To avoid under or over constraining, there is the need for, e.g., an adaptive method in the application of the constraints.
(3) What is the significance of PPP-AR in multi-GNSS PPP atmospheric constrained solution?
  PPP-AR plays a vital role and enables an improved atmospheric-constrained solution. Results presented in this paper with GPS-AR show the greatest improvement at the 95th percentile.
(4) Finally, what are the key challenges left in obtaining near-instantaneous PPP convergence akin to RTK data processing?
  With typical RTK convergence of 10 min, results presented show how close we are to near-instantaneous convergence. With improved atmospheric products, as well as orbits, clocks and bias estimation, PPP solution accuracy is bound to improve.

Future work would involve resolving ambiguities on GPS, Galileo and BeiDou in both dual- and triple-frequency modes while constraining the atmospheric parameters. Given the additional frequencies being transmitted due to modernization and new satellite launches, ambiguity resolution with extra-widelane combinations of multi-frequency observables will also be investigated. The improved multi-GNSS PPP-AR processing technique is also intended to be applied to next-generation low-cost, single- and dual-frequency receivers currently being released. There is also the need for an adaptive approach in the application of correct and realistic ionospheric constraints.

## Figures and Tables

**Table 1:** Processing strategy and parameters used in YorkU GNSS PPP engine for data analysis.

Processing Parameters	YorkU GNSS PPP Engine Settings
Processing technique	Uncombined mode using raw strength of observations
Antenna corrections	IGS ANTEX
Satellite orbits and clocks	GBM (GFZ)
Elevation mask	Minimal 10°
GNSS system	GPS, GLONASS, Galileo and BeiDou
Observation processing mode	Dual-frequency, Triple-frequency, GPS ambiguity resolution, static processing
Data format	RINEX 2.× or 3.×
Ionospheric mitigation	Slant ionospheric delays: estimated GIM delays: used to constrain only the first epoch
Troposphere modelling	Hydrostatic delay: Davis (GPT) Wet delay: estimated Mapping function

**Table 2:** Different existing GIM products available to the PPP user from different ACs [33].

Product	Agency	Type of Product	#Stations	#Satellites	GNSS	Mapping Function
c1pg	AIUB	1-day predicted	~120	~56	*GNSS	NONE
c2pg	AIUB	2-day predicted	~120	~56	*GNSS	NONE
carg	AOE	Post-processed (R)	--	--	*MIX	COSZ
casg	AOE	Post-processed (F)	--	--	*MIX	COSZ
codg	AIUB	Post-processed (F)	~259	~56	*GNSS	NONE
corg	AIUB	Post-processed (R)	~118	~55	*GNSS	NONE
e1pg	ESA/ESOC	1-day predicted	--	--	GPS	NONE
e2pg	ESA/ESOC	1-day predicted	--	--	GPS	NONE
ehrg	ESA/ESOC	Post-processed (R)	~231	~54	GPS	NONE
emrg	NRCAN	Post-processed (R)	~351	~29	GPS	MOD
esag	ESA/ESOC	Post-processed (F)	~300	~54	GPS	NONE
esrg	ESA/ESOC	Post-processed	~236	~54	GPS	NONE
igrg	IGS	Post-processed (CR)	~296	0	*MIX	COSZ
igsg	IGS	Post-processed (CF)	~328	~32	*MIX	COSZ
jplg	JPL	Post-processed (F)	~170	~31	GPS	NONE
jprg	JPL	Post-processed (R)	~170	~31	GPS	NONE
u2pg	UPC	Predicted	--	--	GPS	NONE
uhrg	UPC	Post-processed (R)	~259	~31	*MIX	COSZ
upcg	UPC	Post-processed (F)	~272	~31	GPS	NONE
uprg	UPC	Post-processed (R)	~272	~31	GPS	NONE
uqrg	UPC	Post-processed (R)	~255	~31	GPS	COSZ
whug	WHU	Post-processed (F)	~314	~31	GPS	MSLM

*MIX/*GNSS = GPS and GLONASS satellites. R = rapid, F = final, CR = combined rapid, CF = combined final.

**Table 3:** Improvements in convergence time achieved in comparison to typical convergence periods (to 10 cm horizontal) defined by the mode of PPP processing.

Processing Modes	Convergence in Minutes (Typical)	Convergence in Minutes (Achieved)
Dual-frequency PPP	20	15 [9,42]
Triple-frequency PPP	20	18 [9]
Dual-frequency atmospheric-constrained PPP	~	6
Triple-frequency atmospheric-constrained PPP	~	2
Dual-frequency PPP-AR	15	11 [43]
Dual-frequency atmospheric-constrained PPP-AR	~	<2
